# Risk and protective factors for mental health problems in children and adolescents during the COVID-19 pandemic: results of the longitudinal COPSY study

**DOI:** 10.1007/s00787-024-02604-6

**Published:** 2024-10-29

**Authors:** F. Zoellner, M. Erhart, A.-K. Napp, F. Reiss, J. Devine, A. Kaman, U. Ravens-Sieberer

**Affiliations:** 1 Department of Child and Adolescent Psychiatry, Psychotherapy, and Psychosomatics, Research Division “Child Public Health”, Center for Psychosocial Medicine, University Medical Center Hamburg-Eppendorf, Martinistraße 52, W 29, 20246 Hamburg, Germany; 2 Alice Salomon University of Applied Sciences, Berlin, Germany; 3 Apollon University of Applied Sciences, Bremen, Germany

**Keywords:** Mental health problems, Risk and protective factors, Children and adolescents, Longitudinal, COVID-19, SDQ

## Abstract

The COVID-19 pandemic has had profound effects on the mental health of children and adolescents worldwide, exacerbating existing challenges and introducing new stressors. This paper explores the impact of risk and protective factors on the mental well-being of young individuals during the pandemic. Using data from the German nationwide, population-based, longitudinal COPSY study (*n* = 2,471, girls: 50.0%, age 7–17 years) spanning nearly three years, this study investigates how factors such as gender, age, parental education, parental depressive symptoms, family cohesion, and social support influence mental health. Mental health problems were assessed using the Strengths and Difficulties Questionnaire (SDQ). Latent growth analysis and structural equation modeling were employed to analyze cross-sectional and longitudinal data collected at five measurement points (initial response rate: 46.8%). Findings revealed that boys and younger children are at a higher risk for mental health problems. Additionally, low parental education, single parenthood, parental burden due to the pandemic and parental depressive symptoms were significantly linked with mental health problems in children. Conversely, personal resources, family cohesion, and social support were associated with less symptoms. Family cohesion additionally buffered against the negative impact of parental depressive symptoms. The study underscores the importance of multi-level interventions that consider individual, familial, and societal factors in promoting positive mental health outcomes among children and adolescents during challenging times. Continued research and collaborative efforts are needed to develop evidence-based strategies for supporting the resilience of young individuals in the face of future adversities.

## Introduction

The COVID-19 pandemic has profoundly impacted the life and mental health of children and adolescents worldwide. Young individuals have been confronted with a multitude of stressors and adversities. These challenges encompassed social isolation resulting from lockdowns and social distancing, disruptions of daily routines due to closures of schools and sports facilities, and heightened anxiety stemming from uncertainties about health and economic stability [1]. Long-term and intricate implications on the mental well-being of children and adolescents remain a paramount concern.

Even prior to the pandemic, young people exhibited a high prevalence of mental health problems, solidifying this issue as one of the foremost public health concerns [2, 3]. According to epidemiological research around 18% of German children and adolescents displayed significant mental health problems in pre-pandemic times [4, 5]. This is in line with international studies finding similar prevalence rates [6, 7]. Mental health problems that manifest before the age of 14 years increase the likelihood of psychiatric disorders in adulthood [8, 9]. Consequently, early interventions are of upmost importance, not only from the standpoint of an individual’s well-being but also from a societal and economic perspective. Mental health problems rank among the leading causes of disabilities in Europe and the world, causing a substantial economic burden to society [2, 7].

During the COVID-19 pandemic, the prevalence rates of mental health problems in children and adolescents have significantly increased, as substantiated by a growing body of international literature [1, 10–16]. The German nationwide longitudinal COPSY study (“COvid-19 and PSYchological Health”) has emerged as a crucial monitoring study for evaluating the mental health of children and adolescents throughout almost three years of the pandemic, spanning five assessment points.

The initial two assessments, conducted during the first and second lockdowns in 2020, revealed a significant increase in mental health problems among German youths, nearly doubling compared to pre-pandemic levels [17]. In summer 2021, with eased restrictions, there was a slight improvement. However, the winter of 2021/22 saw a resurgence in mental health problems as COVID cases increased. By the fifth assessment in late 2022, there was a notable decline in mental health problems, though rates remained higher than pre-pandemic levels [17]. The mental burden that children experienced during the pandemic was initially largely overlooked and is still neglected by decision-makers, despite mental health experts urgently calling for interventions [18].

Children do not respond to challenges in the same way. Variability in resilience can be attributed to a variety of factors, including genetic predispositions, familial support, the social environment, individual coping strategies, and past life experiences. Recognizing signs of psychological distress early on and providing appropriate support is crucial, whether within the family, school, or medical support structures. To design tailored prevention and intervention programs, knowledge of the determinants affecting the onset and development of mental health symptoms in young people is vital.

Our pre-pandemic epidemiological research on risk and protective factors for mental health problems in German children and adolescents suggested that younger boys have a higher risk for general mental health problems than younger girls [4, 5]. Further, we found that female gender and adolescent age were associated with higher levels of depressive and anxiety symptoms [19, 20]. Socio-economic disadvantage and parental psychopathology proved to be important risk factors for various mental health outcomes [19–21]. In addition, we found that protective factors, such as self-efficacy, positive family climate, and social support, were associated with less depressive symptoms [19].

During the COVID-19 pandemic, it appears that similar risk and protective factors have influenced mental health outcomes among young people, as indicated by a recent review [1]. Among other determinants, socio-economic disadvantage, parental psychopathology, a dysfunctional family environment, social isolation and loneliness have increased the risk of suffering negative mental health, while self-efficacy, family functioning, contact to friends, and social support served as protective factors [1]. For age and gender the findings were mixed. Female gender served as a risk factor for internalizing symptoms like depression, anxiety, and psychosomatic symptoms, while male gender was a risk factor for general mental health problems, attention problems, and addictive behavior. Younger age was a risk factor for behavioral and emotional problems, and psychosomatic symptoms, as older age was associated with higher rates of depression, anxiety, and addictive behavior [1].

However, there is a research gap studying risk and protective factors across the whole duration of the pandemic, as well as exploring how protective factors interacted during that time. So, the aim of the current study is to extend the existing body of research by investigating enduring, longitudinal effects of risk and protective factors on mental health during the three years of the COVID-19 pandemic (from 2020 to 2022). By exploring the longitudinal effects and interactions, we strive for valuable insights for developing evidence-based prevention and intervention strategies.

Specifically, our research questions are as follows:

- Are male gender and younger age risk factors for mental health problems in children and adolescents at baseline and across the three pandemic years?
- Are low parental education, migration background, and single parenthood risk factors for mental health problems initially and over three years?
- Are parental depressive symptoms and parental burden (due to the pandemic) risk factors for mental health issues at the initial assessment and over time?
- Do personal resources, family cohesion, and social support serve as resource factors against mental health problems at the initial assessment and over time (main effects)?
- Do personal, familial, and social protective factors mitigate the detrimental effect of parental depressive symptoms and parental burden on mental health symptoms in youth (interaction effects)?

Drawing from literature and our prior research, we designate determinants as “risk factors” when they heighten the probability of mental health problems. Conversely, we categorize them as “resource factors” when they correlate with favorable mental health outcomes, or as “protective factors” when they counteract the impact of risk factors [22, 23]. In the literature, risk and resource/protective factors are often categorized into personal, familial, and social factors [23], and we align with this classification.

## Methods

### Study and participants

The German population-based longitudinal COPSY study assessed children, adolescents and one of their parent/caregiver at five time points: At the start of the pandemic, 05–06/2020 (t1, *n* = 1586), in 12/2020-01/2021 (t2, *n* = 1625), in 09–10/2021 (t3, *n* = 1618), in 02/2022 (t4, *n* = 1668), and in 09–10/2022 (t5, *n* = 1701). Families were invited to participate in the survey through an online panel using quota sampling, which ensured that the sample reflected the sociodemographic characteristics of the German population. Families, who had previously participated in the COPSY study, were re-invited for each follow-up. To address drop-outs, achieve sociodemographic representativeness, and maintain comparability across all five waves, additional families were recruited. The initial response rate was 46.8%. On average the respondents participated in 66.3% of the five waves. More details on sampling and inclusion criteria are described elsewhere [24, 25]. Participants aged 7 to 17 years at baseline were included in the current analyses, if data were available for at least one of the five measurement points. This resulted in an overall sample of *N* = 2,471. All measures were assessed by parent-report. Overall, 56.3% of the reporting parents were female.

### Measures

The study included internationally established and validated instruments to assess the mental health of children and adolescents, as well as risk and resource factors.

#### Sociodemographic variables

As sociodemographic variables, we recorded the age and gender of children and adolescents at baseline. The gender categories included “female”, “male”, and “diverse“. Parents reported on their education by two items asking for the highest academic and vocational qualification of both parents. According to the international “Comparative Analysis of Social Mobility in Industrial Nations” (CASMIN) classification, the vocational qualification was categorized on a 10-point scale [26] and then into three status groups (low, medium, and high). Additionally, we assessed information on migration background by two questions and single parenthood by one question.

#### Mental health problems of children and adolescents

Mental health problems of children and adolescents were assessed with the internationally well-established Strengths and Difficulties Questionnaire (SDQ). It contains 20 items with response options from 1 = “not true”, 2 = “somewhat true”, to 3 = “certainly true” [27]. The total score of all items ranges from 0 to 40. Higher scores indicate more severe mental health problems. Cronbach’s α ranged from 0.84 to 0.87 across measurement points.

#### Parental burden due to the pandemic

Parental burden was assessed by a pilot-tested, self-developed single item asking for the perceived overall burden of the pandemic [28] with response options ranging from 1 = “not at all difficult / burdensome” to 5 = “very difficult / burdensome”.

#### Parental depressive symptoms

Parental depressive symptoms were assessed by the eight-item Patient Health Questionnaire (PHQ-8), which is a valid diagnostic and severity measure for depressive disorders [29]. Each item was offered with four response options ranging from 1 = “not at all” to 4 = “nearly every day”. A sum score was calculated with values ranging from 0 to 24. Cronbach’s α ranged from 0.89 to 0.90 across measurement points.

#### Personal resources

Personal resources were measured using five parent-reported items administered also in The German Health Interview and Examination Survey for Children and Adolescents (KiGGS) study [30]. The scale captures individual capabilities such as self-efficacy, optimism, and a positive self-concept. The items (e.g., “My child looks to the future with optimism / confidence”) were provided with four response options (1 = “not true” to 4 = “exactly true”). The whole scale comprises a total score ranging from 5 to 20. Cronbach’s α ranged from 0.81 to 0.86 across measurement points.

#### Family cohesion

Family cohesion was assessed using the corresponding subscale of the parent-report version of the Family Climate Scale (FSC) [31]. It covers feelings of connection and unity among individual members within a family. Each of the four items (e.g., “In our family everybody cares about each other’s worries”) was provided with four-point response options (1 = “not true” to 4 = “exactly true”). The sum score ranges from 4 to 16. Cronbach’s α varied from 0.86 to 0.89 across measurement points.

#### Social support

Data on social support were collected using a short form with four parent-reported items from the German translation of the Social Support Scale (SSS) [32]. The items (e.g., “How often has there been someone your child can count on to listen to when he/she needs to talk”) were answered using five response options (1 = “never” to 5 = “always”). The scale comprises a total value from 4 to 20. Cronbach’s α ranged from 0.83 to 0.86 across measurement points.

### Data analysis

Descriptive analyses were carried out covering frequencies, means, and standard deviations for all variables. Latent growth modeling (LGM) was used to investigate changes in behaviors. Two latent parameters were estimated with the intercept representing the state of a variable at baseline and the slope reflecting the change in this variable over time. We simultaneously calculated LGM for each construct, which was longitudinally measured (i.e., parental depression). The time scores for the slope growth factor were fixed at 0, 1, and 2 to define a linear growth model with equidistant time points. The zero time score for the slope growth factor at baseline defined the intercept growth factor as an initial status factor. The coefficients of the intercept growth factor were fixed at one as part of the growth model parameterization. The residual variances of the outcome variables were estimated and allowed to be different across time. Residuals were not correlated. The intercepts of the outcome variables at the three time points were fixed at zero. The estimator for the parameters was maximum likelihood with robust standard error estimation.

We specified two structural equation models (SEMs): One explaining the intercepts of mental health problems by the intercepts of the other constructs and some covariates. In the other model the slopes of mental health problems were explained by the intercepts and the slopes of the other constructs as well as some covariates. Specifically, we specified direct paths from the intercepts of the risk and resource factors on the intercept and on the slope of mental health problems in children. Direct paths from the slopes of the risk and resources on the slope of mental health problems were also specified and estimated according to the maximum likelihood criterion with robust standard errors. The latent parameters of the risk and resource factors as well as the intercept and the slope of mental health problems were freed to correlate.

Mplus 8 [33] was used for LGMs and for SEM, IBM SPSS 26 for all other analyses.

## Results

The analyzed sample comprised *N* = 2,471 children and adolescents aged 7 to 17 years (M = 12.25, SD = 3.30) at baseline, with 50.0% females, as detailed in Table 1. The majority of parents (56.8%) had a medium level of education. Of all parents, 16.0% had a migration background, and 18.9% were single parents. The mean score for mental health problems in children and adolescents at baseline was M = 9.87 (SD = 6.05). Among the risk factors, parental depressive symptoms yielded a mean score of M = 5.50 (SD = 5.01) and parental burden of 3.22 (SD = 0.95). Regarding resource factors, the mean scores for personal resources were M = 15.37 (SD = 2.70), for family cohesion M = 12.94 (SD = 2.32), and for social support M = 16.33 (SD = 2.85). Means and standard deviations of all measurement points are displayed in Table 1.

**Table 1:** Descriptive data of the analysed sample

	Baseline (t1)(*n* = 1586)	t2 (*n* = 1625)	t3(*n* = 1618)	t4(*n* = 1668)	t5(*n* = 1701)
	n (%)	n (%)	n (%)	n (%)	n (%)
**Sociodemographic factors** ^**1**^					
Female Gender	793 (50.0)	800 (49.3)	823 (51.0)	836 (50.1)	844 (49.6)
Age (in years); M (SD)	12.25 (3.3)	12.69 (3.3)	13.27 (3.3)	12.93 (3.7)	13.06 (4.0)
Low parental education	288 (18.5)	293 (18.4)	289 (18.2)	266 (16.2)	234 (14.0)
Migration background	254 (16.0)	314 (16.5)	355 (17.1)	399 (17.3)	275 (16.1)
Single parenthood	299 (18.9)	287 (17.7)	322 (19.9)	310 (18.6)	301 (16.3)
	**M (SD)**	**M (SD)**	**M (SD)**	**M (SD)**	**M (SD)**
**Risk factors**					
Parental burden	3.22 (0.95)	3.31 (0.93)	3.26 (0.95)	3.25 (0.91)	3.09 (0.97)
Parental depressive symptoms	5.50 (5.01)	6.22 (5.37)	5.90 (5.11)	6.25 (5.15)	5.58 (5.00)
**Resource factors**					
Personal resources	64.25 (17.40)	62.86 (18.35)	64.43 (17.94)	64.65 (17.59)	67.21 (17.19)
Family cohesion	74.35 (17.53)	73.11 (18.12)	74.25 (18.37)	75.15 (17.80)	75.25 (17.91)
Social support	81.64 (15.12)	79.95 (16.77)	81.70 (15.59)	81.11 (15.60)	83.41 (14.57)
**Outcome**					
Mental health problems in children and adolescents	9.87 (6.05)	10.00 (6.45)	9.46 (6.47)	9.67 (6.40)	8.75 (6.10)

^1^ Sociodemographic information were available for the complete sample under analysis (*n* = 1,586). M = mean; SD = standard deviation. For measures see text (Methods)

Intercepts and slopes of the latent growth models are presented in Table 2. Firstly, we looked at the cross-sectional results of the baseline data, which are depicted in the first section of Table 3 under the heading “LGM intercepts”. We found that male gender and younger age were significantly associated with more initial mental health problems. Additionally, low parental education and single parenthood were found to be correlated with higher initial mental health problems in children and adolescents, while migration background was not significantly connected to mental health problems. Regarding risk factors, initial parental burden due to the pandemic and initial parental depressive symptoms were significantly linked with initial mental health problems in youth. Children with more personal resources and a better family cohesion showed fewer mental health problems at baseline. Social support was not found to be a significant resource factor at baseline.

**Table 2:** Intercepts and slopes of latent growth models

Construct (Scale)	Range ofraw scores of measure	Intercept		Slope	
		mean	variance	mean	variance
**Risk factor**					
Parental burden	1–5	3.30	0.54	-0.04	0.12
Parental mental health problems (PHQ-8)	0–24	5.92	19.41	-0.01	0.54
**Resource factors**					
Personal resources	5–20	14.48	4.16	0.12	0.11
Family cohesion (FSC)	4–16	12.87	2.69	0.03	0.05
Social support (SSS)	4–20	17.00	3.55	0.05	0.07
**Outcome**					
Mental health problems in children and adolescents (SDQ)	0–40	10.21	29.29	-0.35	0.77

**Table 3:** Predictors of initial status and change in mental health symptoms of children and adolescents

	Structural equation modelpredicting initial mental healthsymptoms^1^ (LGM intercepts)			Structural equation modelpredicting change in mental health symptoms^2^ (LGM slopes)		
	b	β	*p*	b	β	*p*
**Sociodemographic factors**						
Female gender	-0.535	-0.049	0.001	-0.104	-0.063	0.029
Age	-0.245	-0.180	0.000	-0.025	-0.120	0.000
Low parental education	-0.160	-0.068	0.000	-0.011	-0.030	0.311
Migration background	0.002	0.000	0.993	-0.048	-0.021	0.485
Single parenthood	0.445	0.032	0.037	0.117	0.055	0.072
**Risk factors**						
Initial parental burden (intercept)	-0.424	-0.058	0.071	-0.103	-0.091	0.692
Change in parental burden (slope)				0.471	0.062	0.939
Initial parental depressive problems (intercept)	0.441	0.359	0.000	-0.022	-0.116	0.598
Change in parental depressive problems (slope)				0.604	0.516	0.602
**Resource factors**						
Initial personal resources (intercept)	-1.287	-0.485	0.000	-0.014	-0.034	0.902
Change in personal resources (slope)				1.590	0.486	0.237
Initial family cohesion (intercept)	-0.383	-0.116	0.009	0.021	0.041	0.841
Change in family cohesion (slope)				-1.282	-0.353	0.028
Initial social support (intercept)	-0.102	-0.036	0.378	-0.012	-0.026	0.895
Change in social support (slope)				-2.173	-0.662	0.068

^1^ Structural Equation Model (*n* = 2,125); Model fit: RMSEA = 0.044; CFI = 0.905; BIC = 182026.777; ^2^ Structural Equation Model (*n* = 2125); Model fit: RMSEA = 0.0.045; CFI = 0.901; BIC = 182128.208; b = Unstandardised regression coefficient; β = Standardised regression coefficient

We subsequently examined longitudinal effects in mental health problems among children and adolescents illustrated in Table 3 (“LGM slopes”). We found, that male gender, younger age, and single parenthood but not low parental education and migration background were significantly related to increasing mental health problems. Regarding risk factors, we found that neither initial nor change in parental depressive symptoms or parental burden due to the pandemic were associated with changes in mental health problems in children. No initial resource factor was associated to change in mental health problems, but an increase in family cohesion as well as in social support protected children form developing mental health problems during the pandemic. Change in personal resources was not influencing changes in mental health problems of children.

**Fig. 1:**
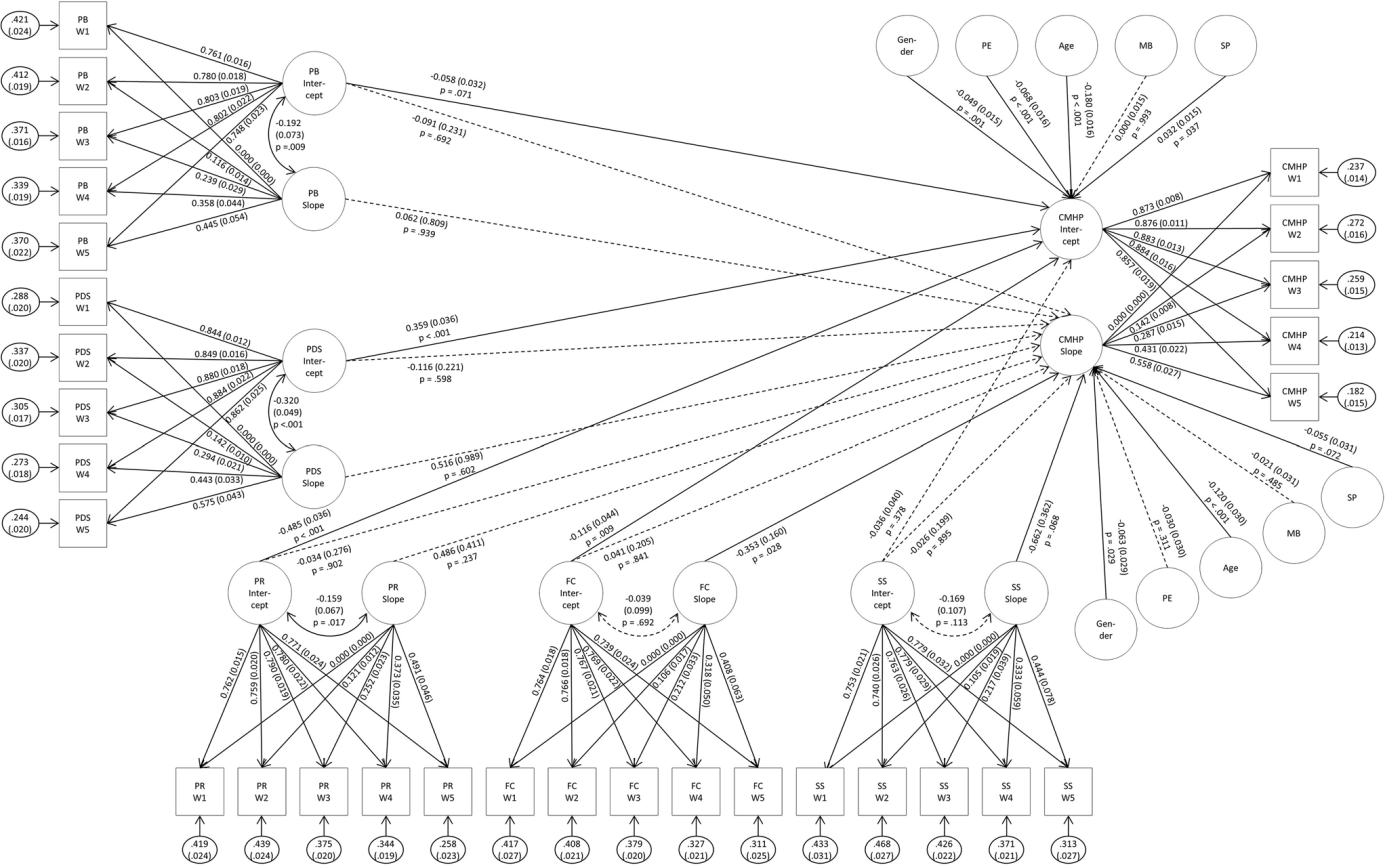
Structural equation model of risk and resource factors of mental health problems in children and adolescents. Standardised estimates (standard errors) are presented, further paths among all intercepts and slopes of risk and resource factors were estimated in the model (not shown for presentation purposes). Dashed lines indicate non-significant effects, continuous arrows indicate significant effects (*p* ≤ 0.1); PE = parental education; MB = migration background; SP = single parenthood; CMHP = child mental health problems; PDS = parental depressive symptoms; PB = parental burden due to the pandemic; PR = personal resources; FC = family cohesion; SS = social support

Figure 1 presents the results of the SEMs. Altogether, using the variables of our baseline model, the model accounted for 67.8% of the variance in the mental health problems intercepts. The variables in our longitudinal model accounted for 70.2% of the variance in the slope of mental health symptoms, however both numbers cannot be compared with the R2 issued from classical linear regression. Please note, that the fit was good for most LGM/SEMs according to the RMSEA (0.044–0.045), but not for the CFI (0.901–0.905) according to Schermelleh-Engel et al. [34].

We tested the potential moderation effect that resource factors may buffer the impact of parental depressive symptoms on child mental health problems in a LGM intercept model including interactions (see Table 4). Indeed, a positive family cohesion did buffer the detrimental effect of parental depressive symptoms on mental health in children at baseline. For personal resources, we found an interaction effect. Surprisingly, we found that increasing social support was associated with a lower relationship between parental depressive problems and mental health problems in children.

**Table 4:** Protective factors moderating the relationship between initial parental depressive symptoms and initial mental health problems in children and adolescents

	Structural equation modelpredicting initial depressive symptoms^1^ (LGM intercept model with interactions)		
	b	β	*p*
**Sociodemographic factors**			
Female gender	-0.525	-0.049	0.001
Age	-0.243	-0.180	0.000
Low parental education	-0.156	-0.067	0.000
Migration background	-0.014	-0.001	0.949
Single parenthood	0.425	0.031	0.046
**Risk factor**			
Initial parental burden (intercept)	-0.392	-0.054	0.098
Initial parental depressive problems (intercept)	0.373	0.306	0.146
**Resource factors**			
Initial personal resources (intercept)	1.209	-0.463	0.000
Initial family cohesion (intercept)	0.067	0.021	0.756
Initial social support (intercept)	-0.533	-0.191	0.002
**Interactions between parental depressive symptoms and protective factors**			
Initial parental depressive symptoms (intercept) by initial personal resources (intercept)	-0.015	-0.026	0.418
Initial parental depressive symptoms (intercept) by initial family cohesion (intercept)	-0.070	-0.095	0.049
Initial parental depressive symptoms (intercept) by initial social support (intercept)	0.069	0.108	0.020

^1^ Structural Equation Model (*n* = 2125); Model fit: BIC = 182032.850; b = Unstandardized regression coefficient; β = Standardized regression coefficient

Due to technical reasons, we could not analyze interaction effects of protective factors on the relationship between parental burden and mental health problems in children. The inclusion of the interaction terms of latent-growth factors addsadd a substantial amount of complexity to the models resulting in non-convergence of the maximum likelihood algorithm. Furthermore, the latent variable covariance matrix was not positive definite. Since the association between risk factors and outcomes was not significant in the structural equation model predicting change in mental health symptoms (LGM slopes), we did not calculate any interaction effects for change in mental health problems as this analysis also was impacted by the above reported issues with the Mplus-Software.

## Discussion

This study examined the cross-sectional and longitudinal impact of risk, resource, and protective factors on the development of mental health problems in children and adolescents throughout the COVID-19 pandemic utilizing a longitudinal growth modeling approach.

We found that boys and younger children were at higher risk for initial and developing mental health problems. Other literature found mixed age and gender differences of poor mental health before and during the pandemic [12]. In their review Wolf and Schmitz [1] report that boys and younger children scored higher in general mental health and attention problems, while girls and adolescents scored higher in internalizing problems, like anxiety and depression. As we assessed general mental health problems using the Strengths and Difficulties Questionnaire [27], known for its focus on externalizing problems, our results are confirming those findings. Our findings advocate for age- and gender-specific prevention and intervention strategies. For example, prevention programs for girls may focus on psychoeducation and coping with anxiety and depressive symptoms including relaxation techniques and anti-rumination techniques, while programs for boys could target hyperactivity, impulse control, and conduct problems using a more behavior-oriented approach. A recent review indicates that most of gender-specific interventions are conducted in schools or community settings [35], however a broad-scale implementation is lacking.

Our study confirms that low parental education and single parenthood are risk factors for children’s mental health [1]. These findings underscore the challenges faced by these families, who often have fewer financial, social, and health resources. Thus, targeted support, including educational initiatives tailored to parents with lower education, are crucial. Additionally, the unique challenges of single-parenthood need to be recognized, and both funding along with social support are essential for children’s well-being. Surprisingly, migration background, a well-established risk factor for mental health problems in German children [36, 37], was not confirmed in our study. This incongruity may stem from the limitation that we could only examine families, who were capable of completing the questionnaire in German. But families proficient in the German language might be more integrated and potentially less affected by disadvantages.

We found that initial parental depressive symptoms and parental burden due to the pandemic had a detrimental effect on the mental health of their children at baseline. This is in line with international literature, finding a significant association between parental and child mental health problems during the pandemic [1, 38]. However, contrary to our assumption, higher baseline parental psychopathology did not predict greater increase in child and adolescent mental health problems longitudinally nor did a change in parental mental health symptoms predict changes in mental health symptoms in children and adolescents. This might be a result of a lack of variation in the slopes of parental and child mental health, because we analyzed a rather healthy general population sample.

In our study, we discovered that higher personal resources, such as optimism and self-efficacy, protected children against mental health problems at the outset of the pandemic. These findings emphasize that personal resources are important for coping at the start of a crisis. However, these resources did not provide additional protection throughout the pandemic. This might be because optimism and self-efficacy are stable personality traits and less likely to change over time.

Family cohesion was an important protective factor, both at baseline and over time. While most of the aforementioned factors are either not modifiable or challenging to change (e.g., gender, age, single parenthood, migration background, parental mental health), family climate is more behavior-dependent and amenable to modification. Encouraging positive interactions, expressing emotions in a healthy manner, and fostering a sense of cohesion can contribute to an improved family climate. Educational, community or counseling programs to enhance parenting skills and support positive family dynamic as well as allocating resources and support to those families are advisable to address the long-term impact of COVID-19 and during future crises.

Social support at baseline was not a resource factor for initial mental health problems. However, we found that an increase in social support over the pandemic was linked to a decrease of mental health problems in children. This suggests that interventions to bolster social support networks for children during times of crisis may help them cope better. Surprisingly, we observed that higher levels of social support were connected to a higher association between mental health problems in parents and children. This finding seemingly contradicts our previous research, where we found social support to be a protective factor for depressive [19] and ADHD symptoms [21] in children. But we encountered a similar paradoxical interaction effect in our study on antisocial behavior [39]. These findings are challenging to interpret and are most likely a methodological artifact, possibly because social support was closely associated with family cohesion. Or one possible interpretation might be that a higher association of parental and child mental health symptoms corresponded with a higher provision of social support (e.g., friends, teachers, educators, neighbors etc.). Suggesting that higher social support could be a consequence rather than a predictor of the association of mental health problems in parents and their children. In future studies we suggest to further investigate whether sustained social support over a longer period can potentially buffer the detrimental effect of parental depressive symptoms on child mental health.

Our results emphasize the importance of multi-level approaches that consider individual, familial, and societal factors. A recent study by Karadzhov et al. (2023) [40] surveyed service providers and policymakers in 22 countries to evaluate effective child well-being practices during COVID-19. The study identified online mental health services, remote learning support, and family outreach programs as key strategies to address children’s needs. After the pandemic, school-based behavior-oriented programs offer an effective and cost-efficient method to reach a majority of children and enhance various short- and long-term mental health outcomes. Programs like “Zippy’s Friends”, designed for preschool and elementary students, have been proven to strengthen coping strategies and reduce mental health difficulties in daily life [41]. Further, providing family support services is crucial in responding to the long-term impact of COVID-19 [42].

This study has several strengths. These include a large population-based sample, a longitudinal design that spanned the entire pandemic, the administration of diverse established questionnaires, and the application of advanced methodology (LGM) on a longitudinal data set. With nearly two decades of experience conducting public health surveys on children’s mental health in Germany and across Europe prior to the pandemic [43–45], we were well-prepared to swiftly plan and execute a survey at the pandemic’s onset. Significant funding efforts allowed us to cover the entire duration of the pandemic, gathering valuable longitudinal data for comparison. Our baseline participation rate was 46%, consistent with other child health surveys [25, 46].

Our study is subject to several limitations. Firstly, changes over time were generally modest, and there was limited variability among individuals due to the general population-based sampling. Secondly, we could not clearly establish a causal relationship between the pandemic and the measured effects, as we did not include pre-pandemic data. Additionally, like all studies, we selected specific risk and protective factors, potentially omitting other relevant ones. Our study findings might be limited by response biases, such as social desirability bias or nonresponse bias. Moreover, potential selection bias may have arisen from our use of exclusively German questionnaires and our results may not be generalizable to countries other than Germany. Finally, we encountered methodological constraints in modeling the LGM as described in the methods section.

## Conclusion

Mental health problems are among the leading causes of disability in the world, often emerging early in life [2, 3, 8]. The COPSY study demonstrated for Germany [17], in line with existing international literature [1, 12], that children’s and adolescents’ mental health has been substantially impaired during the COVID‑19 pandemic.

The findings of our study demonstrate that protective factors, such as family cohesion, can mitigate risk factors and safeguard children’s mental health. Our results emphasize the importance of implementing gender- and age-specific prevention and intervention programs. Additionally, they call for targeted support for children and families with low education levels, single-parent households, and those facing mental health challenges. This support should be provided through funding, educational and parenting programs, and mental health interventions. Raising awareness about the need for mental health support for children and adolescents is crucial across policy, education, and healthcare sectors. In light of challenges such as the pandemic but also other hardships like the climate crises, wars or economic adversities, policymakers should prioritize enhancing screening methods for at-risk youth and providing support to vulnerable youths and parents through prevention and intervention programs.

Continued research and collaborative efforts are needed to develop evidence-based strategies that address mental health promotion in young people in challenging times. Such efforts are crucial for bolstering the resilience of young individuals and equipping them with the tools to navigate future challenges effectively.

## Data Availability

The data that support the findings of this study are available from the corresponding author upon reasonable request.
